# Solitary mandibular recurrence of uterine endometrial carcinoma successfully treated with chemotherapy alone: A case report

**DOI:** 10.1016/j.gore.2025.102013

**Published:** 2025-12-17

**Authors:** Takato Ishida, Shoji Maenohara, Hiroshi Yagi, Hideaki Yahata, Kiyoko Kato

**Affiliations:** Department of Obstetrics and Gynecology, Kyushu University Hospital, Japan

**Keywords:** Endometrial carcinoma, Chemotherapy, Mandibular recurrence, Pembrolizumab

## Abstract

**Background:**

Recurrence of endometrial carcinoma typically occurs in the pelvic region, with the lungs and lymph nodes being common sites for distant metastasis. However, recurrence involving the head and neck region is exceedingly rare.

**Case presentation:**

We report a case of solitary mandibular recurrence of endometrial carcinoma successfully treated with lenvatinib and pembrolizumab. A 56-year-old woman was diagnosed with stage II clear cell carcinoma of the uterus. She underwent total abdominal hysterectomy, bilateral adnexectomy, partial omentectomy, appendectomy, pelvic and *para*-aortic lymphadenectomy. Postoperative adjuvant chemotherapy with six cycles of paclitaxel and carboplatin was administered, followed by observation. Two months after the completing of treatment, the patient developed trismus and pain in the left mandible. Imaging studies revealed a 4 cm mass in the left mandible. Needle biopsy confirmed a solitary recurrence of endometrial carcinoma. After the administration of pembrolizumab and lenvatinib therapy, the tumor was no longer detectable, which has been maintained.

**Conclusion:**

Mandibular metastasis from endometrial carcinoma is extremely rare and may mimic primary jaw tumors. Treatment options include surgical resection, radiotherapy, and systemic therapy. A comprehensive evaluation of the primary tumor status, presence of other metastases, and the patient’s functional condition is essential for determining the optimal therapeutic strategy.

## Highlights

• Solitary mandibular metastasis from uterine clear cell carcinoma is extremely rare.
• We presented lenvatinib and pembrolizumab therapy achieved complete response without surgery or radiotherapy.
• Mandibular pain and trismus may indicate metastasis in gynecologic cancer patients.

## 1 Introduction

According to the latest reports on endometrial carcinoma in Japan, the 5-year survival rate is 94.1 % for stage I and 88.8 % for stage II, indicating good outcomes and a relatively low risk of recurrence (Kajiyama et al., 2025). It has been reported that half of the recurrence sites for endometrial carcinoma are confined to the pelvic region, while distant metastases are often found in the lungs and lymph nodes (Aalders et al., 1984). Recurrences with metastasis to the head and neck are rare, but have been described in sporadic case reports.

Herein, we report a rare case of stage II endometrial clear cell carcinoma that developed a solitary recurrence in the mandibular bone. The patient was treated with lenvatinib and pembrolizumab (LP) therapy and had a favorable clinical course.

## 2 Case presentation

A 56-year-old nulligravida woman presented with abnormal uterine bleeding and was referred to our department for further evaluation following a cervical cytology result of atypical glandular cells and a cervical biopsy raising the suspicion of adenocarcinoma. Endometrial biopsy performed at our institution confirmed clear cell carcinoma. Pelvic contrast-enhanced MRI suggested invasion of more than half of the myometrium, and the lesion was localized to the uterus. Contrast-enhanced CT scan of the neck, chest, and pelvis showed no distant metastasis or lymphadenopathy. The patient was diagnosed with endometrial carcinoma, and had a total abdominal hysterectomy, bilateral adnexectomy, partial omentectomy, appendectomy, pelvic lymphadenectomy, and *para*-aortic lymphadenectomy.

In the postoperative pathology, clear cell carcinoma was identified, with myometrial invasion of 8/18 mm, positivity for stromal invasion in the cervix, negativity for vascular invasion, and no lymph node metastasis, leading to a diagnosis of stage II endometrial carcinoma (FIGO 2008). Six courses of carboplatin (AUC = 6) and paclitaxel (175 mg/m^2^) (TC) therapy were administered every 3 weeks. Subsequent CT scans showed no signs of recurrence, and the patient was placed under observation.

**Fig. 1:**
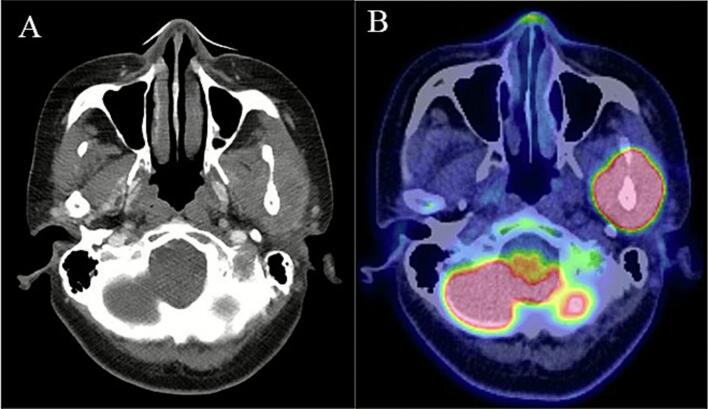
Pretreatment imaging findings A: Contrast-enhanced CT reveals a mass in the left mandible. B: PET/CT shows intense uptake corresponding to the left mandibular mass.

**Fig. 2:**
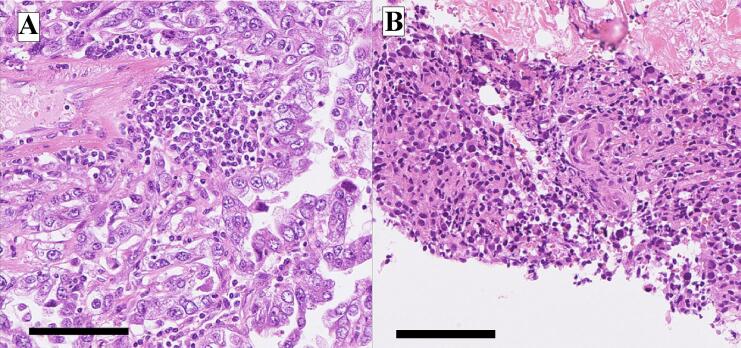
Histopathological findings A: In the HE staining of the initial surgical tissue, atypical cells with clear cytoplasm are proliferating. B: In the biopsy tissue, the atypical cells are morphologically similar to the tumor cells of known endometrial carcinoma, which is consistent with recurrence. Black bar indicates 100 μm.

**Fig. 3:**
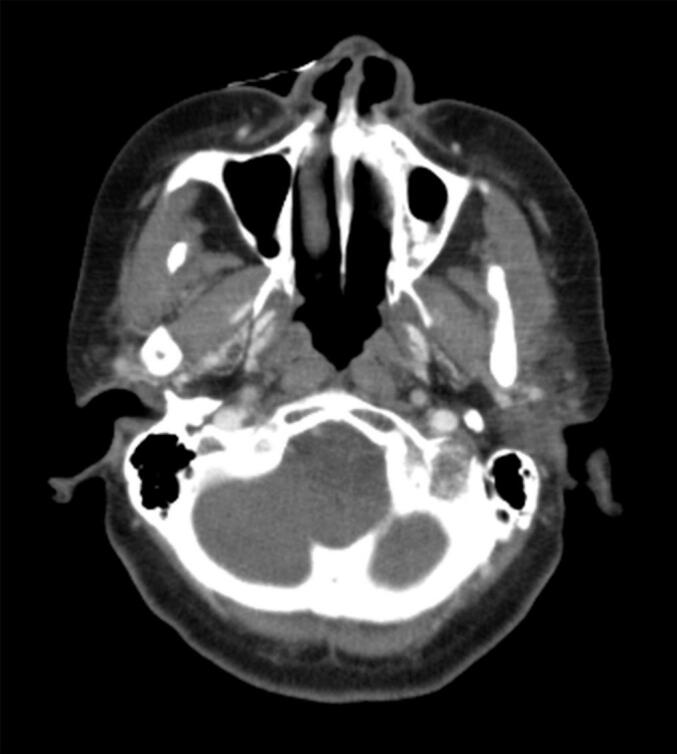
Post-treatment imaging findings CT scan performed 4 months after the initiation of LP therapy shows no detectable lesion in the left mandible, consistent with complete response.

Approximately 2 months after the completion of chemotherapy, the patient developed gradually progressive trismus and left mandibular pain. She visited a local dental clinic and was diagnosed with temporomandibular joint disorder, but the symptoms did not improve over time. Five months after the completion of chemotherapy, CT scan of the jaw was performed, revealing a mass in the left mandible. She revisited our hospital for further evaluation and treatment. CT scan showed a 4 cm mass in the left mandible, associated with bone destruction (Fig. 1A). There were no obvious distant metastases or lymphadenopathy in other organs. PET-CT scan showed abnormal uptake in the tumor of the left mandible (Fig. 1B). In needle biopsy of the mandibular tumor, the morphology of the atypical cells was similar to that in the primary uterine tumor (Fig. 2). Immunohistochemical staining showed positivity for AE1/AE3, p53, and HNF1β, focal positivity for Napsin A, and negativity for ER and PgR, consistent with recurrence of uterine clear cell carcinoma. The patient was diagnosed with recurrence of endometrial carcinoma with a pMMR status, and treatment with lenvatinib and pembrolizumab was started. Her trismus and mandibular pain improved for four weeks after initiating therapy. During the course of treatment, grade 2 uveitis, generalized fatigue, and grade 2 rash were observed. Owing to the patient’s strong wishes, lenvatinib was discontinued after the third course of pembrolizumab. After the third cycle of pembrolizumab, CT imaging showed tumor shrinkage, and no detectable lesion was observed after the sixth cycle (Fig. 3). The response was assessed as a complete response according to RECIST criteria. From the fifth cycle onward, the dosing regimen was changed to 400 mg every six weeks. As of manuscript submission, the cumulative dose had reached 3200 mg/body, and treatment has since continued for over a year without any signs of recurrence.

## 3 Discussion

Recurrence of endometrial carcinoma typically occurs within the pelvis, but when limited to the clear cell carcinoma subtype, two-thirds of recurrences occur outside the pelvis, with the most common sites being the upper abdomen, liver, and lungs, while bone metastasis is rare (Abeler et al., 1996).

Meanwhile, among malignant neoplasms occurring in the oral cavity, metastatic tumors are rare, accounting for about 1 % of the total, with many reports documenting the primary sites as the lung and breast (Hirshberg et al., 1994). However, metastases from gynecological organs have also been reported. According to a review by Irani et al., in 453 cases of mandibular metastasis (including 245 men and 208 women), breast carcinoma (81 cases), thyroid carcinoma (34 cases), and lung carcinoma (17 cases) were the most commonly observed in women. Gynecological carcinomas accounted for 15 cases (Irani, 2017). Hirshberg et al. also reported that, among 390 patients with mandibular metastasis, 11 had a primary uterine tumor (Hirshberg et al., 1994).

The mandible is the most common site of oral metastasis, possibly due to the tortuous course and slow blood flow of the inferior alveolar artery (Banerjee, 1967), as well as the abundance of red marrow, which may facilitate tumor cell seeding (van der Kwast and van der Waal, 1974). The most common symptoms of malignant tumors of the mandible are pain and swelling there; however, if there is also pain in the temporomandibular joint and limitation of mouth opening, the symptoms may resemble those of temporomandibular joint disorders. In patients with similar symptoms who have a history of malignant tumors, or who do not respond to treatment for temporomandibular joint pain, the possibility of metastatic tumors in the mandible should also be considered (Cai et al., 2016).

Metastatic tumors in the oral cavity are often discovered as one of multiple distant metastases. Treatment options include surgical resection, radiation therapy, and chemotherapy, but owing to the poor prognosis, palliative care is also a viable option (Hirshberg et al., 1994). Despite advances in mandibular reconstruction, postoperative complications such as taste disturbance, trismus, rehabilitation challenges, and esthetic concerns remain (Bak et al., 2010). Radiotherapy may lead to mucositis, infection, reduced salivary secretion, fibrosis, sensory deficits, dental caries, and osteonecrosis (Sroussi et al., 2017). Given these risks, treatment planning should be based on comprehensive assessment of primary tumor control, presence of other metastases, and the patient’s activities of daily living.

In recent years, it has been reported that the combination therapy of lenvatinib and pembrolizumab in advanced or recurrent endometrial carcinoma outperformed systemic chemotherapy in all aspects, including progression-free survival, overall survival, and overall response rate (Makker et al., 2023). In the PEACOCC study, Kristeleit et al. reported that 98 % (45 out of 46) of clear cell gynecologic cancers were pMMR, and demonstrated clinical benefit of pembrolizumab in this population. Notably, six of the 46 cases were endometrial carcinomas (Kristeleit et al., 2025). This case involved a solitary recurrence at the mandible. Although surgery and radiation therapy were considered, we chose chemotherapy given the risk of decreased quality of life from treatment-related complications and the favorable outcomes of LP therapy. Additionally, since recurrence occurred soon after TC therapy and the patient had a status of mismatch-repair proficient, we chose LP therapy. Regarding mandibular metastasis of endometrial carcinoma, Maxymiw et al. reported a case in which radiation therapy was administered, but there were also multiple lymph node metastases and lumbar spine metastasis (Maxymiw and Wood, 1991). Additionally, Dosoretz et al. reported a case of solitary recurrence in the right mandible in endometrial carcinoma (Dosoretz et al., 1999). After administering six courses of radiation therapy and TC chemotherapy, the mandibular tumor initially disappeared. However, recurrences were observed in the left mandible as well as in the epidural space and the pelvic cavity. Surgery was performed for the epidural tumor, and radiation therapy was conducted for the pelvic recurrence (Bak et al., 2010). To the best of our knowledge, this case represents the first report of treatment for solitary mandibular metastasis of endometrial carcinoma solely with chemotherapy.

## 4 Conclusion

We experienced a case of solitary recurrence of endometrial carcinoma in the mandible. While solitary metastasis to the mandible is extremely rare in gynecologic carcinomas, if there is no response to treatment for temporomandibular joint pain during the follow-up of gynecologic carcinoma, the possibility of metastatic tumors in the mandible should be considered. To choose the most appropriate treatment, it is necessary to comprehensively assess the control status of the primary lesion, the presence of other metastatic lesions, activities of daily living, and other factors. However, LP therapy is considered advantageous for quality of life and should be regarded as a viable option.

## Consent

Written informed consent was obtained from the patient for publication of this case report and accompanying images. A copy of the written consent form is available for review by the Editor-in-Chief of this journal on request.

## CRediT authorship contribution statement

**Takato Ishida:** Writing – original draft, Investigation. **Shoji Maenohara:** Writing – original draft, Investigation, Conceptualization. **Hiroshi Yagi:** Writing – review & editing. **Hideaki Yahata:** Writing – review & editing. **Kiyoko Kato:** Writing – review & editing.

## Declaration of competing interest

The authors declare that they have no known competing financial interests or personal relationships that could have appeared to influence the work reported in this paper.

## References

[b0005] Aalders J.G., Abeler V., Kolstad P. (1984). Recurrent adenocarcinoma of the endometrium: a clinical and histopathological study of 379 patients. Gynecol. Oncol..

[b0010] Abeler V.M., Vergote I.B., Kjorstad K.E., Trope C.G. (1996). Clear cell carcinoma of the endometrium. Prognosis and Metastatic Pattern Cancer.

[b0015] Bak M., Jacobson A.S., Buchbinder D., Urken M.L. (2010). Contemporary reconstruction of the mandible. Oral Oncol..

[b0020] Banerjee S.C. (1967). Metastasis to the mandible. Oral Surg. Oral Med. Oral Pathol..

[b0025] Cai Z., Zhu C., Wang L., Zhu L., Zhang Z., Zhu H. (2016). A retrospective study of six patients with mandibular metastatic carcinoma. Oncol. Lett..

[b0030] Dosoretz D.E., Orr J.W., Salenius S.A., Orr P.F. (1999). Mandibular metastasis in a patient with endometrial cancer. Gynecol. Oncol..

[b0035] Hirshberg A., Leibovich P., Buchner A. (1994). Metastatic tumors to the jawbones: analysis of 390 cases. J. Oral Pathol. Med..

[b0040] Irani S. (2017). Metastasis to the Jawbones: a review of 453 cases. J. Int. Soc. Prev. Community Dent..

[b0045] Kajiyama, H., Tamauchi, S., Takahashi, F., Kawana, K., Board members of the Committee on Gynecologic Oncology of the Japan Society of, O., Gynecology, 2025. Annual report of the committee on gynecologic oncology, the Japan Society of Obstetrics and Gynecology: Annual patient report for 2021 and annual treatment report for 2016. J. Obstet. Gynaecol. Res. 51, e16168. DOI: 10.1111/jog.16168. 10.1111/jog.16168 39587792

[b0050] Kristeleit R., Devlin M.J., Clamp A., Gourley C., Roux R., Hall M. (2025). Pembrolizumab in patients with advanced clear cell gynecological cancer: a phase 2 nonrandomized clinical trial. JAMA Oncol..

[b0055] Makker V., Colombo N., Casado Herraez A., Monk B.J., Mackay H., Santin A.D. (2023). Lenvatinib plus pembrolizumab in previously treated advanced endometrial cancer: updated efficacy and safety from the randomized phase III study 309/KEYNOTE-775. J. Clin. Oncol..

[b0060] Maxymiw W.G., Wood R.E. (1991). Metastatic endometrial carcinoma to the mandible: a case report. J. Oral Maxillofac. Surg..

[b0065] Sroussi H.Y., Epstein J.B., Bensadoun R.J., Saunders D.P., Lalla R.V., Migliorati C.A. (2017). Common oral complications of head and neck cancer radiation therapy: mucositis, infections, saliva change, fibrosis, sensory dysfunctions, dental caries, periodontal disease, and osteoradionecrosis. Cancer Med..

[b0070] van der Kwast W.A., van der Waal I. (1974). Jaw metastases. Oral Surg. Oral Med. Oral Pathol..

